# Urinary NGAL and RBP Are Biomarkers of Normoalbuminuric Renal Insufficiency in Type 2 Diabetes Mellitus

**DOI:** 10.1155/2019/5063089

**Published:** 2019-09-15

**Authors:** Aimei Li, Bin Yi, Yan Liu, Jianwen Wang, Qing Dai, Yuxi Huang, Yan Chun Li, Hao Zhang

**Affiliations:** ^1^Department of Nephrology, The Third Xiangya Hospital, Central South University, Changsha, 410013 Hunan, China; ^2^Department of Medicine, Division of Biological Sciences, The University of Chicago, Chicago, IL 60637, USA

## Abstract

**Objectives:**

As a screening index of diabetic kidney disease (DKD), urinary albumin/creatine ratio (UACR) is commonly used. However, approximately 23.3%-56.6% of DKD patients with estimated glomerular filtration rate (eGFR) < 60 ml/min per 1.73 m^2^ are normoalbuminuric. Thus, urinary biomarkers of nonalbuminuric renal insufficiency in type 2 diabetes mellitus (T2DM) patients are urgently needed.

**Methods:**

This cross-sectional study enrolled 209 T2DM patients with normoalbuminuria whose diabetes duration was more than 5 years. The patients were classified into two groups, NO-CKD (eGFR ≥ 60 ml/min per 1.73 m^2^, *n* = 165) and NA-DKD (eGFR < 60 ml/min per 1.73 m^2^, *n* = 44). Levels of urinary neutrophil gelatinase-associated lipocalin (NGAL), retinol-binding protein (RBP), plasminogen activator inhibitor-1 (PAI-1), vascular cell adhesion molecule-1 (VCAM-1), and E-cadherin were detected, and their correlations with eGFR, plasma TNF-*α*, IL-6, endothelin-1 (ET-1), and 8-hydroxydeoxyguanosine (8-OHdG) were assessed.

**Results:**

Among patients with renal insufficiency, 26.0% was normoalbuminuric. Compared to the NO-CKD group, the NA-DKD group was older with lower hemoglobin (HB) levels and higher systolic blood pressure (SBP), plasma TNF-*α*, IL-6, and 8-OHdG levels. Logistic regression analysis suggested that age, TNF-*α*, and 8-OHdG were independent risk factors for nonalbuminuric renal insufficiency. Compared to the NO-CKD group, the NA-DKD group exhibited significant increases in urinary NGAL and RBP levels but not PAI-1, VCAM-1, and E-cadherin. Urinary NGAL and RBP both correlated negatively with eGFR and positively with plasma IL-6 and 8-OHdG. Multiple linear regression indicated NGAL (*β* = −0.287, *p* = 0.008) and RBP (*β* = −44.545, *p* < 0.001) were independently correlated with eGFR.

**Conclusion:**

Age, plasma TNF-*α*, and 8-OHdG are independent risk factors for renal insufficiency in T2DM patients with normoalbuminuria. Urinary NGAL and RBP can serve as noninvasive biomarkers of normoalbuminuric renal insufficiency in T2DM.

## 1. Introduction

Renal insufficiency, one of the main microvascular complications of diabetes, is characterized by chronic inflammation and is associated with a substantially increased risk of mortality [1]. Due to the rising incidence of diabetes, diabetic kidney disease (DKD) has become the primary cause of chronic kidney disease (CKD) among the elderly population in China [2].

DKD is defined as albuminuria (urinary albumin/creatine ratio (UACR) > 30 mg/g), an impaired glomerular filtration rate (GFR) (GFR ≤ 60 ml/min per 1.73 m^2^), or both [3]. As the noninvasive of urine test, elevated urinary albumin level is considered an early index of DKD. However, approximately 23.3%-56.6% of patients with T2DM and kidney function decline have normal albuminuria [4–8]. It has been reported that among T2DM patients with renal insufficiency, all-cause mortality in the normoalbuminuric group is not significantly lower than that in the proteinuric group [9]. Moreover, normoalbuminuric renal insufficiency is a strong predictor of mortality in individuals with T2DM [10]. However, there is no sensitive urinary biomarker for normoalbuminuric renal insufficiency in T2DM.

Compared to T2DM patients with albuminuria, patients with a decreased GFR and normoalbuminuria are more likely to be older and have lower HbA1c and diabetic retinopathy prevalence and higher prevalence of coronary heart disease [6, 11, 12]. The classical pathological changes in diabetic nephropathy include thickening of the glomerular basement membrane, mesangial expansion, nodular sclerosis, global glomerular sclerosis, arteriolar hyalinosis, and interstitial fibrosis [13, 14]. However, a study conducted by Ekinci et al. [15] found that in T2DM patients with reduced eGFR, compared with 22 of 23 patients with albuminuria, only 3 of 8 patients with normoalbuminuria had typical glomerular changes. Nonetheless, compared with only 1 of 23 patients with albuminuria, 3 of 8 patients with normoalbuminuria had predominantly interstitial or vascular changes. Moreover, varying degrees of arteriosclerosis are observed in 7/8 subjects with normoalbuminuria. These findings suggest that tubulointerstitial damage and macroangiopathy may be involved in the development of normoalbuminuric renal insufficiency.

Numerous risk factors are known to contribute to the pathogenesis of DKD, including inflammation, endothelial dysfunction, oxidative stress, and epigenetic regulations [16–18], while the pathogenesis of normoalbuminuric renal insufficiency remains unclear. Studies have supported elevated TNF-*α* and IL-6 levels participated in kidney damage of DM [19, 20]. Expression of endothelin-1 (ET-1) was elevated in the kidney of STZ-treated rat diabetic model [21]. Meanwhile, high level of plasma 8-hydroxydeoxyguanosine (8-OHdG) was associated with increased risk of kidney disease in DM patients [19, 20, 22–24]. In the current study, we analyzed the relationship between plasma TNF-*α*, IL-6, ET-1, and 8-OHdG with eGFR in T2DM patients with normoalbuminuria and evaluated the role of indicators of tubulointerstitial damage (neutrophil gelatinase-associated lipocalin (NGAL), retinol-binding protein (RBP), plasminogen activator inhibitor-1 (PAI-1), vascular cell adhesion molecule-1 (VCAM-1), and E-cadherin) in the urine as potential biomarkers for normoalbuminuric renal insufficiency in T2DM.

## 2. Methods

### 2.1. Study Participants

We recruited 432 Chinese T2DM patients (diagnosed according to the 1999 World Health Organization (WHO) criteria), including 169 patients with declined kidney function (eGFR < 60 ml/min per 1.73 m^2^), from the Department of Nephrology and Endocrinology of the Third Xiangya Hospital of Central South University between September 2016 and December 2018. All patients involved in this study fulfilled the following inclusion criteria: age > 18 years old; initial diagnosis of diabetes ≥ 5 years ago; no fever, infection, or trauma; not undergoing surgery or dialysis; no acute diabetic complications (such as diabetic ketoacidosis or nonketone hypertonic coma); and no severe cardiovascular or cerebrovascular diseases in the 3-6 months before recruitment. Patients with normoalbuminuria (UACR < 30 mg/g, *n* = 209) were divided into NO-CKD (eGFR > 60 ml/min per 1.73 m^2^, *n* = 165) group and NA-DKD (eGFR < 60 ml/min per 1.73 m^2^, *n* = 44) group. eGFR was calculated based on the CKD-EPI-combined creatinine-cystatin C equation which appears to be the optimal GFR estimate method [25]. Informed consent was obtained from all participants before they participated in the study. This study adhered to the Declaration of Helsinki and was approved by the Ethics Committee of the Third Xiangya Hospital of Central South University.

### 2.2. Laboratory Measurements

All urine and blood samples were obtained from the patients in the morning after 12 h of fasting on the first day of their hospitalization. Patient medical history and anthropometric measurements were recorded on the same day. The urine samples were stored at -80°C after being centrifuged for 15 min at 2000×g. The blood samples, which were collected in tubes with K_2_EDTA, were used for hemoglobin A1c (HbA1c) analysis. The serum was separated, aliquoted, and used for routine chemical tests. Hemoglobin (HB) was measured using an automatic blood cell analyzer (Sysmex, Japan). Total cholesterol (TC), triglyceride (TG), high-density lipoprotein (HDL), low-density lipoprotein (LDL), serum albumin (ALB), fasting blood sugar (FBS), plasma creatinine (CR), blood urea nitrogen (BUN), uric acid (UA), cystatin C (CYSC), urinary creatinine, urinary microalbumin, and urinary RBP levels were measured by an automatic biochemistry analyzer (Hitachi, Japan). HbA1c levels were measured by an automatic glycosylated hemoglobin analyzer (Bio-Rad, USA). Urinary NGAL, PAI-1, and VCAM-1 levels were measured by magnetic Luminex assays using human premixed multianalyte kits (R&D Systems, USA), and urinary E-cadherin and plasma ET-1, TNF-*α*, IL-6, and 8-OHdG levels were measured by enzyme-linked immunosorbent assays (ELISAs) using commercially available standard kits (R&D Systems, Abcam, CUSABIO, USA).

### 2.3. Statistical Analyses

SPSS version 16.0 (Chicago, IL, USA) and RStudio (Boston, MA, USA) were used for statistical analyses. Data are expressed as the mean ± SD for normally distributed values or the median (25-75th percentiles) for nonparametric values, and categorical variables are expressed as ratios. PAI-1, E-cadherin, TNF-*α*, and 8-OHdG values were logarithmically transformed. All urinary biomarkers were normalized to urinary creatinine, and the square root was then calculated for analyses. UACR values were Napierian logarithmically transformed. Pearson's or Spearman's correlation coefficients were calculated to assess the associations between biomarkers and eGFR. Multiple linear regression was used to analyze relationships among clinical parameters, urinary biomarkers, and eGFR. Logistic regression analysis was performed to identify risk factors for eGFR. Differences between groups were analyzed by ANOVA, followed by LSD's test for normally distributed values, the Kruskal-Wallis test for nonparametric values, or the chi-square test for categorical variables. Receiver operating characteristic (ROC) curves were drawn to calculate the area under the ROC curve (AUC). A very good test would have an AUC > 0.9, a good test would have an AUC of 0.7-0.9, a sufficient test would have an AUC of 0.5-0.7, and a faulty test would have an AUC ≤ 0.5. All reported *p* values are two-tailed, a *p* value < 0.05 was considered statistically significant, and a *p* value < 0.01 was considered highly significant.

## 3. Results

### 3.1. Patients' Baseline Characteristics

Among the 432 T2DM patients, 209 patients were normoalbuminuric, and 223 were albuminuric. Their clinical characteristics are presented in [Supplementary-material supplementary-material-1]. Compared with patients with normoalbuminuria, the levels of urinary NGAL, RBP, PAI-1, VCAM-1, and E-cadherin were increased in patients with albuminuria, and all correlated positively with UACR ([Supplementary-material supplementary-material-1]). Our results were consistent with previous studies [26–30]. Because nonalbuminuric renal insufficiency in T2DM patients may be ignored clinically and there is no sensitive urinary biomarker, we focused on T2DM patients with nonalbuminuria in this study.

Among the 209 T2DM patients with normoalbuminuria, there were 165 patients without renal insufficiency (eGFR > 60 ml/min per 1.73 m^2^, NO-CKD group) and 44 patients with renal insufficiency (eGFR < 60 ml/min per 1.73 m^2^, NA-DKD group). 26.0% (44/169) of patients with renal insufficiency was normoalbuminuric. Compared to the NO-CKD group, the NA-DKD group had higher age, SBP, BUN, CR, UA, and CYSC levels and lower HB level (Table 1).

### 3.2. Correlations between Plasma TNF-*α*, IL-6, 8-OHdG, and ET-1 with eGFR in T2DM Patients with Normoalbuminuria

**Figure 1:**
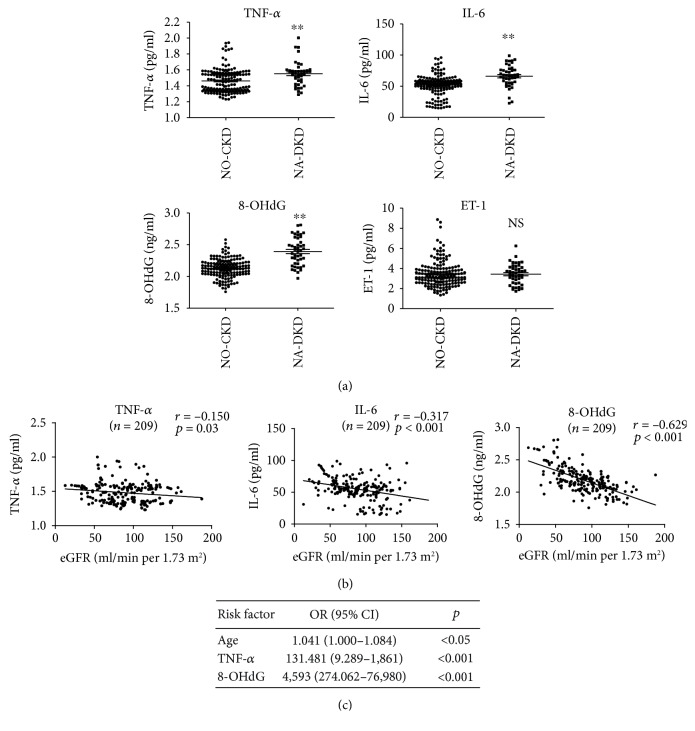
Correlations between plasma TNF-*α*, IL-6, 8-OHdG, and ET-1 with eGFR in T2DM patients with normoalbuminuria. (a) Differences in the plasma TNF-*α*, IL-6, 8-OHdG, and ET-1 levels in patients with normoalbuminuria with/without renal insufficiency. (b) Relationships of plasma TNF-*α*, IL-6, and 8-OHdG with the eGFR in T2DM patients with normoalbuminuria. (c) Logistic regression analyses between clinical parameters, plasma TNF-*α*, IL-6, and 8-OHdG and eGFR. 8-OHdG: 8-hydroxydeoxyguanosine; ET-1: endothelin-1; NO-CKD: normoalbuminuric T2DM patients without renal insufficiency; NA-DKD: normoalbuminuric T2DM patients with renal insufficiency; eGFR: estimated glomerular filtration rate. ^∗∗^*p* < 0.01 vs. the NO-CKD group. NS: no significant.

Inflammation, endothelial dysfunction, and oxidative stress are known to contribute to the pathogenesis of DKD [16, 17]. To explore the roles of inflammation, endothelial dysfunction, and oxidative stress in normoalbuminuric renal insufficiency, we examined levels of plasma TNF-*α*, IL-6, ET-1, and 8-OHdG in 209 T2DM patients with normoalbuminuria. The plasma TNF-*α*, IL-6, and 8-OHdG levels were higher in the NA-DKD group than in the NO-CKD group, while the plasma ET-1 level was not different between these two groups (Figure 1(a)). Single linear regression analysis showed that plasma TNF-*α*, IL-6, and 8-OHdG levels were each negatively correlated with eGFR (*r* = −0.150, *p* = 0.03; *r* = −0.317, *p* < 0.001; and *r* = −0.629, *p* < 0.001, respectively) (Figure 1(b)). After adjusting for potential confounding factors (age, SBP, and HB), logistic regression analyses revealed that age (odds ratio (OR) = 1.041, 95% CI 1.000-1.084, *p* < 0.001), TNF-*α* (OR = 131.481, 95% CI 9.289-1,861, *p* = 0.048), and 8-OHdG (OR = 4.593, 95% CI 274.062-76,980, *p* < 0.001) were independent risk factors for renal insufficiency in T2DM patients with normoalbuminuria (Figure 1(c)).

### 3.3. Correlations between Urinary NGAL, RBP, PAI-1, VCAM-1, and E-Cadherin with eGFR in T2DM Patients with Normoalbuminuria

**Figure 2:**
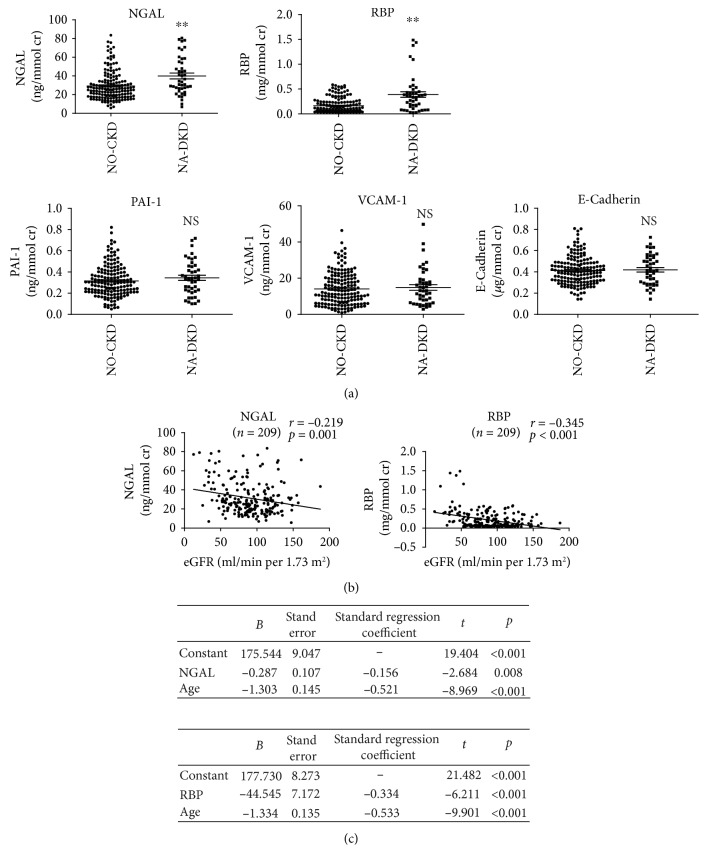
Correlations between urinary NGAL, RBP, PAI-1, VCAM-1, and E-cadherin with eGFR in T2DM patients with normoalbuminuria. (a) Levels of urinary NGAL, RBP, PAI-1, VCAM-1, and E-cadherin in patients with normoalbuminuria with/without renal insufficiency. (b) Single linear regression analysis of urinary NGAL, RBP, and eGFR in T2DM patients with normoalbuminuria. (c) Multiple linear regression analysis of urinary NGAL or RBP, clinical parameters, and eGFR. NGAL: neutrophil gelatinase-associated lipocalin; RBP: retinol-binding protein; PAI-1: plasminogen activator inhibitor-1; VCAM-1: vascular cell adhesion molecule-1. ^∗∗^*p* < 0.01 vs. NO-CKD group. NS: no significant.

Compared to NO-CKD group, levels of urinary NGAL and RBP but not PAI-1, VCAM-1, or E-cadherin were significantly elevated in NA-DKD group (Figure 2(a)). Single linear regression analysis revealed that urinary NGAL and RBP had negative correlations with eGFR (*r* = −0.291, *p* = 0.001 and *r* = −0.345, *p* < 0.001, respectively) in the study patients (Figure 2(b)). Moreover, multiple linear regression analyses showed that after adjusting for potential confounding factors (age, SBP, and HB), either NGAL or RBP and age were independently associated with eGFR (*Y*_eGFR_ = 175.544 − 0.287 NGAL − 1.303 age; *Y*_eGFR_ = 177.730 − 44.545 RBP − 1.334 age) (Figure 2(c)).

### 3.4. Correlations between Plasma TNF-*α*, IL-6, ET-1, and 8-OHdG with Urinary NGAL and RBP

**Figure 3:**
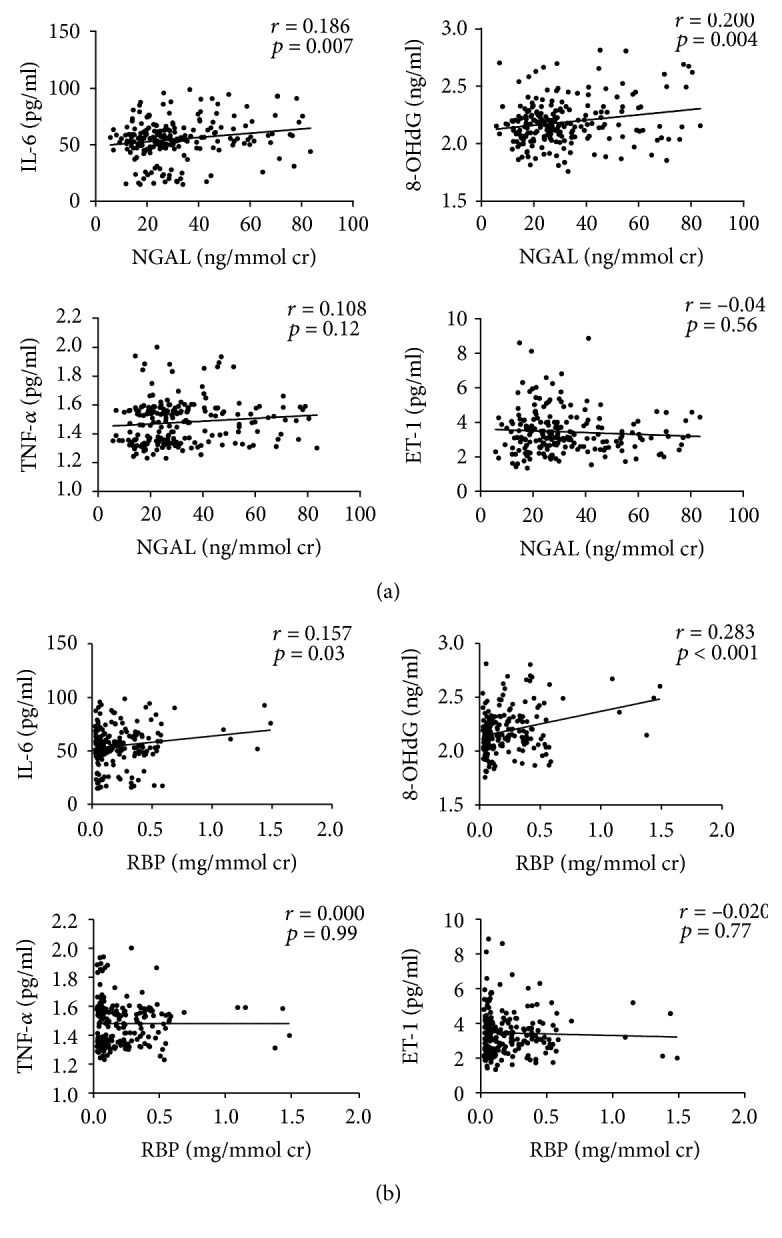
Correlations between plasma TNF-*α*, IL-6, ET-1, and 8-OHdG with urinary NGAL and RBP. (a) Single linear regression analysis of plasma TNF-*α*, IL-6, ET-1, and 8-OHdG and urinary NGAL. (b) Single linear regression analysis of plasma TNF-*α*, IL-6, ET-1, and 8-OHdG and urinary RBP.

To identify the possible reason for the increase in NGAL and RBP levels in T2DM patients with normoalbuminuric renal insufficiency, we performed single linear regression analysis on the relationships between plasma TNF-*α*, IL-6, ET-1, and 8-OHdG with urinary NGAL and RBP in T2DM patients with normoalbuminuria. As shown in Figure 3(a), positive associations were found between NGAL and IL-6 (*r* = 0.186, *p* = 0.007) and NGAL and 8-OHdG (*r* = 0.200, *p* = 0.004). In addition, RBP was positively associated with IL-6 (*r* = 0.157, *p* = 0.023) and 8-OHdG (*r* = 0.283, *p* < 0.001) (Figure 3(b)), while there were no significant associations between NGAL or RBP and TNF-*α* or ET-1.

### 3.5. ROC Analyses of Urinary NGAL and RBP for Renal Insufficiency in T2DM Patients with Normoalbuminuria

**Figure 4:**
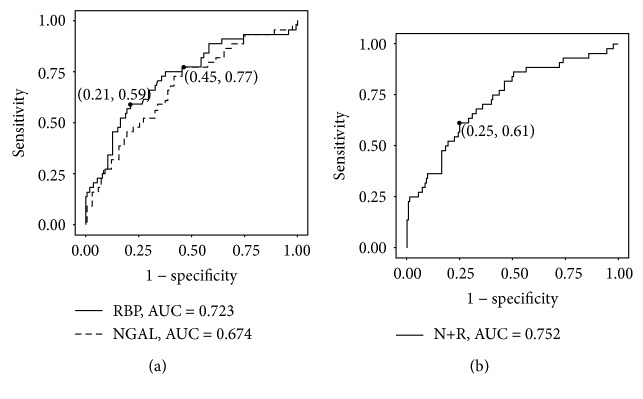
ROC analyses of urinary NGAL and RBP for renal insufficiency in T2DM patients with normoalbuminuria. (a) ROC curve for the ability of NGAL and RBP to identify cases with eGFR < 60 ml/min per 1.73 m^2^ among patients with normoalbuminuria. (b) ROC curve for the combined detection of urinary NGAL and RBP (N+R) to identify cases with eGFR < 60 ml/min per 1.73 m^2^ among patients with normoalbuminuria. ROC: receiver operating characteristic; AUC: area under the ROC curve; N+R: combined detection of urinary NGAL and RBP.

Next, we analyzed the diagnostic accuracy of urinary NGAL and RBP for renal insufficiency in T2DM patients with normoalbuminuria via ROC curves. The results indicated that urinary NGAL had sufficient diagnostic accuracy (sensitivity = 0.773, specificity = 0.545, AUC = 0.674) and that RBP had good diagnostic accuracy (sensitivity = 0.591, specificity = 0.788, AUC = 0.723) for eGFR < 60 ml/min per 1.73 m^2^ in T2DM patients, but the difference between urinary NGAL and RBP was not significant (*p* = 0.32) (Figure 4(a)). Next, we analyzed the diagnostic accuracy of combined detection of NGAL and RBP. The ROC curve showed that combined detection had good diagnostic accuracy (sensitivity = 0.614, specificity = 0.752, AUC = 0.731) (Figure 4(b)), but there was no significant difference between combined detection and NGAL alone (*p* = 0.13) or RBP alone (*p* = 0.66).

### 3.6. Characteristics of Propensity Score-Matched Cohorts

**Figure 5:**
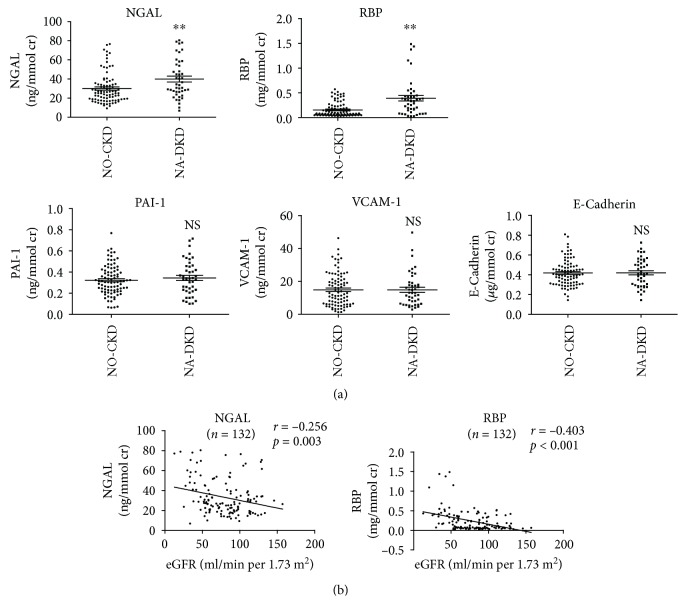
Correlations between urinary NGAL, RBP, PAI-1, VCAM-1, and E-cadherin with eGFR in matched T2DM patients with normoalbuminuria. (a) Levels of urinary NGAL, RBP, PAI-1, VCAM-1, and E-cadherin in matched patients with normoalbuminuria with/without renal insufficiency. (b) Single linear regression analysis of urinary NGAL, RBP, and eGFR in matched T2DM patients with normoalbuminuria. ^∗∗^*p* < 0.01 vs. the NO-CKD group. NS: no significant.

Because age was independently associated with eGFR in T2DM patients with normoalbuminuric renal insufficiency, to better control for confounding factors, we used propensity score matching to compare urinary biomarkers between the NO-CKD and NA-DKD groups. Following the 2-to-1 matching by propensity score, 88 patients in the NO-CKD group were matched to 44 patients in the NA-DKD group. Among the matched patients, compared to the NO-CKD group, the NA-DKD group had higher SBP, BUN, CR, UA, and CYSC, but no difference in age and HB (Table 2). Compared to the NO-CKD group, in age-matched patients, the levels of urinary NGAL and RBP but not PAI-1, VCAM-1, or E-cadherin were significantly elevated in the NA-DKD group (Figure 5(a)). Similar to unmatched data, urinary NGAL and RBP showed negative correlations with eGFR (*r* = −0.256, *p* = 0.003 and *r* = −0.403, *p* < 0.001, respectively) (Figure 5(b)). These results indicate that urinary NGAL and RBP can serve as biomarkers in age-matched T2DM patients with normoalbuminuric renal insufficiency.

## 4. Discussion

In this study, we found that age, plasma TNF-*α*, and 8-OHdG were independent risk factors for renal insufficiency in T2DM patients with normoalbuminuria. In addition, levels of urinary NGAL and RBP were elevated in patients with renal insufficiency and negatively related to eGFR in T2DM patients with normoalbuminuria. Furthermore, urinary NGAL and RBP were independently associated with eGFR, suggesting that urinary NGAL and RBP are biomarkers for T2DM patients with normoalbuminuric renal insufficiency.

Diabetic kidney disease is the most common cause of ESRD, the primary clinical characteristics of which are progressive proteinuria and renal failure [31]. However, a portion of patients with T2DM follows a nonalbuminuric pathway to renal impairment that may be misdiagnosed. A previous cross-sectional survey including 301 T2DM patients showed that the prevalence of GFR < 60 ml/min per 1.73 m^2^ and normoalbuminuria was 23.3% [6], which is highly consistent with the prevalence in our study. Studies have reported that aging, rising blood pressure, and intrarenal vascular disease may play pathogenic roles in nonalbuminuric renal insufficiency [6] [15], which might explain why the patients with nonalbuminuric renal insufficiency in our study had higher age and SBP values than others.

Inflammation appears to associated with renal function decline in normoalbuminuric renal insufficiency [32]. Our previous study confirmed that the level of TNF-*α* was increased in T2DM patients [33], and the current study showed levels of plasma TNF-*α* and IL-6 were elevated in T2DM patients with renal insufficiency and were inversely related to the eGFR. Generally, diabetic nephropathy is believed to be a microvascular complication of DM, while studies have indicated that macroangiopathy was more prevalent in patients with normoalbuminuric renal insufficiency, which was usually accompanied by significant cardiovascular disease burden [8, 11, 12]. As a sensitive biomarker of intracellular oxidative stress, 8-OHdG was shown to be more highly expressed in a high intima media thickness (IMT) group than in a normal IMT group, and 8-OHdG was also correlated positively with coronary heart disease risk scores [34], suggesting that 8-OHdG is a useful biomarker of macrovascular complications in patients with T2DM. Our results showed that 8-OHdG was an independent risk factor for renal insufficiency in T2DM patients with normoalbuminuria. ET-1 is a potent vasoconstrictor mainly produced by mesangial cells in the kidneys, and renal microcirculation is particularly susceptible to ET-1 [35], which may explain why plasma ET-1 levels were found to be elevated in diabetic patients with microalbuminuria [36], but not elevated in our patients with normoalbuminuric renal insufficiency. These results indicate that inflammation and oxidative stress may be factors contributing to the pathogenesis of normoalbuminuric renal insufficiency, which seems more relevant to macroangiopathy of T2DM.

In diabetic patients with proteinuria, the increase in interstitial fibrosis correlates with renal function decline, and this decline is independent of albuminuria, indicating that interstitial injury partly contributes to the declining eGFR in DM [37]. In patients with normoalbuminuric renal insufficiency, interstitial or vascular changes are observed more frequently than the renal structural change typical of diabetic nephropathy [15], which suggests that tubulointerstitial damage may be involved in the development of normoalbuminuric renal insufficiency. NGAL and RBP, tubular damage biomarkers, have low molecular weights which are filtered by glomeruli and reabsorbed by proximal tubules [38]. Injured tubules reduce reabsorption and thus increase their urinary excretion in T2DM patients with normoalbuminuric renal insufficiency. In previous studies, NGAL expression increased in patients with T1DM before the diagnosis of microalbuminuria [39] and increased progressively from UACR < 10 mg/g to 10-30 mg/g to >30 mg/g in T2DM patients [40]. Urinary RBP had been identified as a biomarker of proximal tubular dysfunction and had good diagnostic value in diabetic patients with macroalbuminuria [41]. Our present study showed that levels of NGAL and RBP were increased in T2DM patients with normoalbuminuric renal insufficiency and weakly but significantly correlated with eGFR, which may be caused by inflammation and oxidative stress, which can cause tubular damage [42]. Both urinary NGAL and RBP are correlated positively with plasma IL-6 and 8-OHdG in our study. Although tubulointerstitial damage is common with older age [43], our study showed urinary NGAL and RBP were elevated in patients with declined kidney function and correlated negatively with eGFR in age-matched patients.

Our study has some limitations. First of all, this cross-sectional study only provided the basis for associations, and a longitudinal study is needed to confirm the value of these biomarkers. Besides, eGFR was calculated based on CKD-EPI-combined creatinine-cystatin C equation, which is not the “gold standards.” In addition, further mechanism research is necessary to verify the roles of NGAL and RBP in normoalbuminuric renal insufficiency.

Taken together, our results reveal that urinary NGAL and RBP are biomarkers for normoalbuminuric renal insufficiency in T2DM, which may be caused by inflammation and oxidative stress. It is necessary to detect urinary NGAL and RBP in T2DM patients, especially elderly individuals.

## Figures and Tables

**Table 1:** Clinical characteristics of T2DM patients with normoalbuminuria.

	NO-CKD (*N* = 165)	NA-DKD (*N* = 44)	*p* value
Age (years)	57.4 ± 12.1	66.4 ± 11.7	<0.001
Gender (male/female)	107/58	28/16	0.861
Diabetes duration (years)	9.9 ± 5.4	10.9 ± 6.2	0.269
BMI (kg/m^2^)	24.15 ± 3.76	23.25 ± 3.07	0.147
SBP (mmHg)	129.8 ± 17.7	136.8 ± 20.9	0.028
DBP (mmHg)	79.9 ± 11.3	82.2 ± 13.2	0.253
HB (g)	132.7 ± 21.3	121.2 ± 26.7	0.003
HbA1C (%)	8.20 (6.85-10.0)	8.00 (7.33-9.00)	0.834
FBS (mmol/l)	8.18 ± 3.12	7.98 ± 3.28	0.714
TC (mmol/l)	4.33 ± 1.35	4.10 ± 1.33	0.310
TG (mmol/l)	1.43 (0.92-2.47)	1.67 (0.98-2.50)	0.654
HDL (mmol/l)	1.10 ± 0.33	1.06 ± 0.32	0.389
LDL (mmol/l)	2.07 ± 0.77	2.04 ± 0.79	0.828
ALB (g/l)	38.1 ± 6.6	37.7 ± 6.3	0.717
BUN (mmol/l)	5.17 (4.39-6.64)	8.21 (6.72-10.61)	<0.001
CR (mmol/l)	72.0 (60.0-84.0)	121.0 (86.0-169.5)	<0.001
UA (*μ*mol/l)	313.4 ± 103.6	393.1 ± 139.6	<0.001
CYSC (mg/l)	0.76 (0.61-0.92)	1.40 (1.26-1.67)	<0.001

**Table 2:** Clinical characteristics of the matched patients.

	NO-CKD (*N* = 88)	NA-DKD (*N* = 44)	*p* values
Age (years)	63.5 ± 10.5	66.4 ± 11.7	0.141
Gender (male/female)	56/32	28/16	0.578
Diabetes duration (years)	10.9 ± 6.1	10.9 ± 6.2	0.968
BMI (kg/m^2^)	23.10 ± 3.51	23.25 ± 3.07	0.803
SBP (mmHg)	129.4 ± 17.0	136.8 ± 20.9	0.033
DBP (mmHg)	78.8 ± 10.6	82.2 ± 13.2	0.108
HB (g)	129.9 ± 15.8	121.2 ± 26.7	0.051
HbA1C (%)	8.15 (6.90-10.0)	8.00 (7.33-9.00)	0.839
FBS (mmol/l)	8.24 ± 3.25	7.98 ± 3.28	0.667
TC (mmol/l)	4.21 ± 1.10	4.10 ± 1.33	0.621
TG (mmol/l)	1.36 (0.85-2.48)	1.67 (0.98-2.50)	0.447
HDL (mmol/l)	1.06 ± 0.27	1.06 ± 0.32	0.902
LDL (mmol/l)	2.05 ± 0.82	2.04 ± 0.79	0.944
ALB (g/l)	37.5 ± 5.5	37.7 ± 6.3	0.868
BUN (mmol/l)	5.55 (4.62-7.02)	8.21 (6.72-10.61)	<0.001
CR (mmol/l)	72.5 (60.0-84.0)	121.0 (86.0-169.5)	<0.001
UA (*μ*mol/l)	310.2 ± 105.0	393.1 ± 139.6	<0.001
CYSC (mg/l)	0.81 (0.64-0.93)	1.40 (1.26-1.67)	<0.001

## Data Availability

The data used to support the findings of this study are included within the article.

## Abbreviations

T2DM: Type 2 diabetes mellitus
DN: Diabetic nephropathy
NGAL: Neutrophil gelatinase-associated lipocalin
RBP: Retinol-binding protein
PAI-1: Plasminogen activator inhibitor-1
VCAM-1: Vascular cell adhesion molecule-1
ESRD: End-stage renal disease
UACR: Urinary albumin/creatinine ratio
EMT: Epithelial-mesenchymal transition
eGFR: Estimated glomerular filtration rate
HB: Hemoglobin
HbA1c: Hemoglobin A1c
FBS: Fasting blood glucose
TC: Total cholesterol
TG: Triglyceride
HDL: High-density lipoprotein
LDL: Low-density lipoprotein
ALB: Serum albumin
BUN: Blood urea nitrogen
CR: Creatinine
UA: Uric acid
CYSC: Cystatin C
8-OHdG: 8-Hydroxydeoxyguanosine
ET-1: Endothelin-1
ROC: Receiver operating characteristic
AUC: Area under the ROC curve
BMI: Body mass index
SBP: Systolic blood pressure
DBP: Diastolic blood pressure
IMT: Intima media thickness.

## Supplementary Materials

Supplementary Materials Table S1: clinical characteristics of the T2DM patients. Figure S1: associations between urinary biomarkers with UACR in T2DM patients. A: levels of urinary biomarkers in T2DM patients with/without albuminuria. B: relationship between urinary biomarkers and UACR in T2DM patients. ^∗∗^*p* < 0.01 vs. the normoalbuminuria group. NGAL: neutrophil gelatinase-associated lipocalin; RBP: retinol-binding protein; PAI-1: plasminogen activator inhibitor-1; VCAM-1: vascular cell adhesion molecule-1.
