# Supplementary material for: Urinary NGAL and RBP Are Biomarkers of Normoalbuminuric Renal Insufficiency in Type 2 Diabetes Mellitus
Source: J Immunol Res. 2019 Sep 15;2019:5063089. doi: 10.1155/2019/5063089 (PMC6766169; doi:10.1155/2019/5063089)
Supplement: Supplementary Materials — Table S1: clinical characteristics of the T2DM patients. Figure S1: associations between urinary biomarkers with UACR in T2DM patients. A: levels of urinary biomarkers in T2DM patients with/without albuminuria. B: relationship between urinary biomarkers and UACR in T2DM patients. ∗∗p < 0.01 vs. the normoalbuminuria group. NGAL: neutrophil gelatinase-associated lipocalin; RBP: retinol-binding protein; PAI-1: plasminogen activator inhibitor-1; VCAM-1: vascular cell adhesion molecule-1. [file 5063089.f1.docx]

**Supplemental Data**

Table S1 Clinical characteristics of the T2DM patients

|  | **Normoalbuminuria**  **(N=209)** | **Albuminuria**  **(N=223)** | **p-value** |
| --- | --- | --- | --- |
| **Age (years)** | 59.3±12.6 | 60.2±11.0 | <0.001 |
| **Gender (male/female)** | 135/74 | 134/89 | 0.193 |
| **Diabetes duration (years)** | 10.0±5.6 | 11.6±6.0 | 0.006 |
| **BMI (kg/m^2^)** | 23.96±3.63 | 24.57±3.57 | 0.078 |
| **SBP (mmHg)** | 131.3±18.6 | 148.6±21.5 | <0.001 |
| **DBP (mmHg)** | 80.4±11.7 | 84.3±12.1 | 0.001 |
| **HB (g)** | 130.3±22.9 | 111.9±25.5 | <0.001 |
| **HbA1C (%)** | 8.2 (6.9-9.8) | 7.7 (6.4-9.7) | 0.022 |
| **FBS (mmol/l)** | 8.13±3.15 | 7.68±3.31 | 0.149 |
| **TC (mmol/l)** | 4.28±1.35 | 4.76±1.59 | 0.001 |
| **TG (mmol/l)** | 1.48 (0.95-2.51) | 1.53 (1.04-2.31) | 0.483 |
| **HDL (mmol/l)** | 1.09±0.33 | 1.13±0.32 | 0.231 |
| **LDL (mmol/l)** | 2.06±0.77 | 2.47±1.03 | <0.001 |
| **ALB (g/l)** | 38.0±6.5 | 34.6±7.4 | <0.001 |
| **BUN (mmol/l)** | 5.70 (4.56-7.53) | 8.27 (5.88-11.26) | <0.001 |
| **CR (mmol/l)** | 77.0 (63.0-96.0) | 114.0 (79.0-279.0) | <0.001 |
| **UA (µmol/l)** | 330.2±116.4 | 393.0±111.5 | <0.001 |
| **CYSC (mg/l)** | 0.86 (0.64-1.12) | 1.32 (0.86-2.45) | <0.001 |


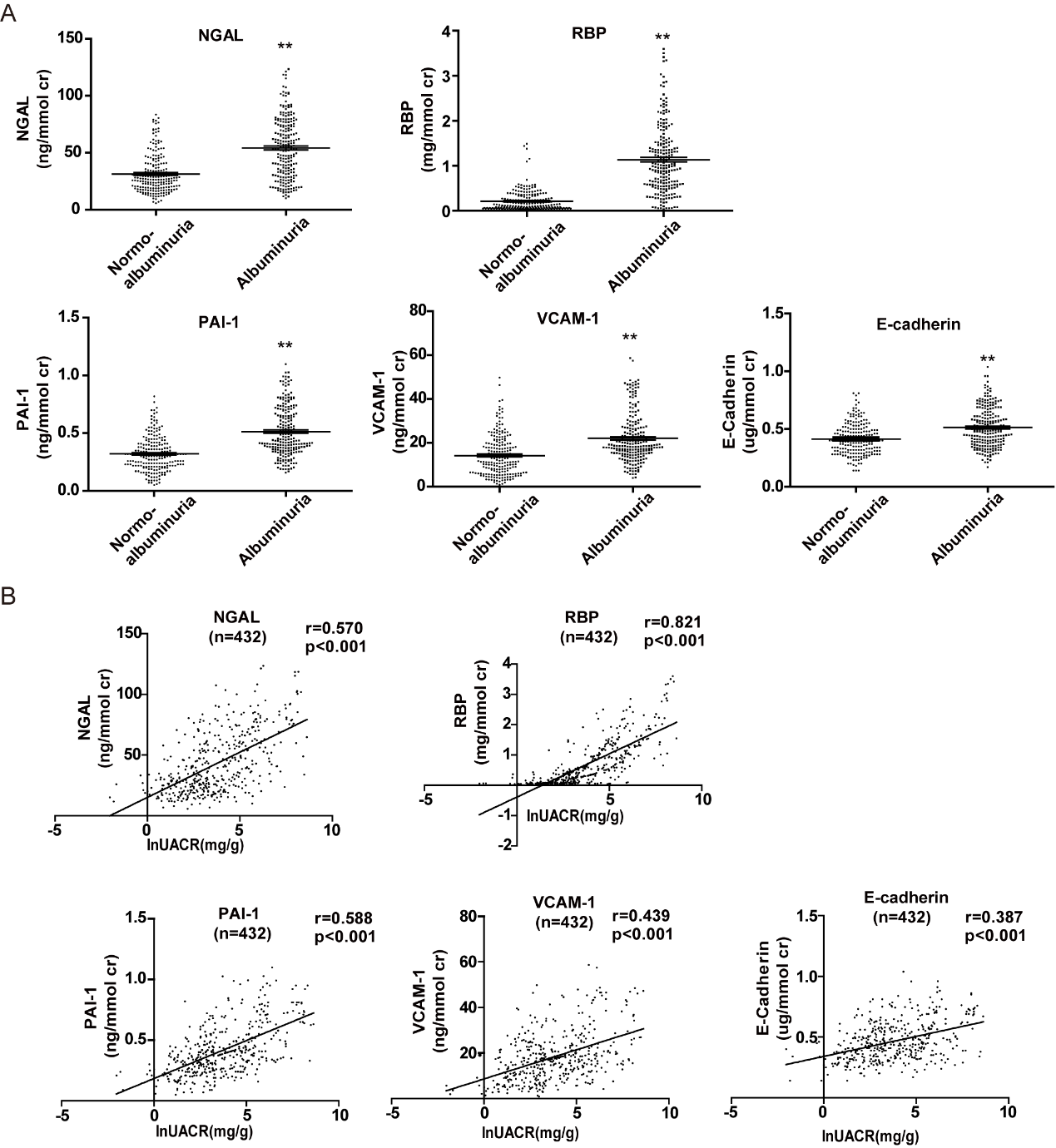


**Fig. S1** Associations between urinary biomarkers with UACR in T2DM patients

A: Levels of urinary biomarkers in T2DM patients with/without albuminuria. B: Relationship between urinary biomarkers and UACR in T2DM patients. **: p＜0.01 vs the Normoalbuminuria group. NGAL: neutrophil gelatinase-associated lipocalin; RBP: retinol binding protein; PAI-1: plasminogen activator inhibitor-1; VCAM-1: vascular cell adhesion molecule-1.
